# Influence of Butyrate Loaded Clinoptilolite Dietary Supplementation on Growth Performance, Development of Intestine and Antioxidant Capacity in Broiler Chickens

**DOI:** 10.1371/journal.pone.0154410

**Published:** 2016-04-22

**Authors:** Yanan Wu, Yanmin Zhou, Changhui Lu, Hussain Ahmad, Hao Zhang, Jintian He, Lili Zhang, Tian Wang

**Affiliations:** College of Animal Science and Technology, Nanjing Agricultural University, Nanjing, Jiangsu, People’s Republic of China; University of Illinois at Chicago, UNITED STATES

## Abstract

The study was conducted to evaluate the effects of dietary butyrate loaded clinoptilolite (CLI-B) on growth performance, pancreatic digestive enzymes, intestinal development and histomorphology, as well as antioxidant capacity of serum and intestinal mucosal in chickens. Two hundred forty 1-day-old commercial Arbor Acres broilers were randomly assigned to 4 groups: CON group (fed basal diets), SB group (fed basal diet with 0.05% sodium butyrate), CLI group (fed basal diet with 1% clinoptilolite), and CLI-B group (fed basal diet with 1% CLI-B). The results showed that supplementation of CLI-B significantly decreased (P < 0.05) feed conservation ratio at both 21 and 42 days of age, improved the pancreatic digestive enzymes activities (P < 0.05), increased the villus length and villus/crypt ratio (P < 0.05), and decreased the crypt depth of intestine (P < 0.05) as compared to the other experimental groups. Furthermore, the CLI-B environment improved the antioxidant capacity by increasing the antioxidant enzyme activities (P < 0.05) in intestine mucosal, and decreasing the NO content and *i*NOS activity (P < 0.05) in serum. In addition, CLI-B supplementation had improved the development of intestine and antioxidant capacity of broilers than supplementation with either clinoptilolite or butyrate sodium alone. In conclusion, 1% CLI-B supplementation improved the health status, intestine development and antioxidant capacity in broiler chickens, thus appearing as an important feed additive for the poultry industry.

## Introduction

Butyrate, one of three main short chain fatty acids (acetate, propionate and butyrate), is gaining more attention for controlling gut pathogens and promote the health status of animals in the animal feed industry. It is produced by the bacterial fermentation of unabsorbed carbohydrates in the colonic lumen of monogastric mammals and birds [1], serves as one of the primary sources of energy for the gastrointestinal epithelium and promotes the growth of normal epithelial cells [2]. Because of its antibacterial effect, butyrate can improve the balance of the intestinal microbiome [3], and has been widely used as a feed additive in the poultry industry [4]. However, butyrate was typically used in the form of sodium butyrate due to the unpleasant odor and a recognizable volatility property of natural butyrate [5]. Oral supplementation of butyrate can enter the systemic circulation and cause a significant increase in the plasma butyrate concentration of both chickens and mice [6, 7]. However, poor pharmacological properties of butyrate, such as first-pass hepatic clearance and short half-life, limited the amount of butyrate that can enter the systemic circulation. Additionally, high doses of butyrate are usually needed to achieve therapeutic concentrations *in vivo* [8]. Meanwhile, non-protected butyrate has been found to only affect the upper part of the digestive tract but not the entire gastrointestinal tract [9, 10]. Therefore, it is necessary to develop a carrier for natural sodium butyrate to overcome these disadvantages.

Clinoptilolite, one of the rarest natural zeolite especially in China, is a crystalline microporous aluminosilicate of alkali and alkaline earth cations with channels and pores running through the crystal [11]. The biological effects of clinoptilolite, such as adsorptivity, cation-exchange, and catalytic properties, are related to its unique structural characteristics, which are known as molecular sieves. Clinoptilolite is currently used in different technological applications such as purification of water, soil improvement, cleaning of fish pond, food supplement and radioprotection etc. It is relatively stable in the gastrointestinal tract of animals [12], and can adsorb heavy metals, free radicals as well as toxins in the body and eventually excrete them from the body as a unique selective adsorbent [13]. The adsorptive characteristics of clinoptilolite affects tissue uptake and the utilization of NH_4_^+^, Cs^+^, Cu^2+^, Cd^2+^, and Pb^2+^ as well as other cations in animals [13, 14]. Thus, clinoptilolite could potentially improve feed efficiency, enhance immunity [15, 16] and reduce oxidant stress in animals [17, 18]. Therefore, clinoptilolite is widely used as a feed additive in the animal industry. The other prospective use of clinoptilolite, as a drug carrier [19, 20], is to control the release time of drugs and keep the concentration of drugs relatively stable over a period of time [21]. Additionally, the structural and adsorption properties of clinoptilolite can be improved through different modification methods such as acid, alkali, heat treatments and microwave modifications. Acid treatment, as the most common modification method, exchanges the cations of the clinoptilolite with H^+^ and can remove aluminum from the framework [22] and improve the adsorption capacity of clinoptilolite by dissolving the impurities that block the pores of clinoptilolite [23]. Therefore, we created an additive that combines the advantages of both materials, butyrate loaded clinoptilolite (CLI-B). We hypothesized that loading butyrate into clinoptilolite would increase the adsorption capacity of clinoptilolite while clinoptilolite could be a carrier for butyrate.

Broiler chicken is one of the most common target species for butyrate administration, but whether CLI-B would improve development and antioxidant capacity of intestine in broilers remains to be elucidated. Therefore, in the present study, we aimed to determine the effects of CLI-B on the growth performance, digestive enzymes activity of pancreas, development of intestine, intestinal histomorphology and antioxidant capacity of serum and intestinal mucosal in broiler chickens.

## Materials and Methods

### Preparation of butyrate loaded clinoptilolite

The clinoptilolite was purchased from the Center of China Geological Survey (Nanjing, China) and sieved through a 100-mesh sieve. CLI-B was synthesized using a sol-gel intercalation [24]. It was then calcined in a muffle oven at 350°C for 2 h to remove the water and organic template to free the pores. Butyrate (Chemical Pure, 98%) and sodium butyrate (Chemical Pure, 98%) were purchased from Aladdin Industrial Corporation (Shanghai, China). Butyrate loaded clinoptilolite was prepared according to the method of Wu et al with some modifications [24]. The clinoptilolite was added to a 70-mL butyrate solution with a concentration of 3 mol/L. The mixture was blended at 60°C at 151 rpm/min in a constant temperature oscillated instrument for 4 h, and the lower sediments were washed by deionized water until the pH of the washed solution was 7. Finally, the washed material was collected and dried at 105°C for 2 h in an air oven and then ground and sieved through a 100-mesh sieve. The temperature (105°C) was much lower than the boiling point of butyrate (163.5°C) which will not affect the function of butyrate, besides, the temperature (105°C) will help to remove the water in clinoptilolite which met the demand of differential thermal analysis (DTA). The clinoptilolite was successfully loaded with butyrate using this method. The butyrate loaded into the clinoptilolite was 3.8%, as determined by a DTA method [25]. Thus, the amount of butyrate in 0.05% SB is the same as that in 1% CLI-B.

### Experimental design, diets and management

Two hundred and forty 1-d-old commercial Arbor Acres broilers were procured from a local commercial hatchery (Hewei, Anhui, China) and were randomly divided into four experimental groups. Each treatment had six replicates and each replicate contained ten birds with similar average body weight and were fed diets as follows: basic diet (CON group), basic diet + 0.05% sodium butyrate (SB group), basic diet + 1% clinoptilolite (CLI group), basic diet + 1% butyrate loaded clinoptilolite (CLI-B group). The basic diet of broilers in all treatments was a corn-soybean basal diet and was formulated based on the NRC (1994). Ingredients and nutrient composition of broiler diets at 21 d and 42 d are available in the Table A in S1 File.

All birds were placed in wired cages and housed in an environmentally controlled room. Temperature was maintained at 34°C to 36°C during 1 to 14 d of age and gradually decreased to 26°C, after which it was held at room temperature and remained unchanged until the end of the experiment. The light regimen was a 12 h light-dark cycle (0600 to 1800 h light). The birds were allowed to consume feed and water *ad-libitum*. This project was approved and conducted under the supervision of the Animal Care and Use Committee, Nanjing Agricultural University, Nanjing, China, which has adopted the Animal Care and Use Guidelines governing all animals used in experimental procedures.

### Sample collection and procedures

One bird was randomly selected from each replicate and weighed after 12 h of feed deprivation at 21 d and 42 d of age. All birds were sacrificed by exsanguination and necropsied immediately. Blood samples were collected without anticoagulant for serum separation. Blood was centrifuged at 4000 rpm for 15 min at 4°C to collect serum. The pancreas tissue samples were collected then cut into pieces. All of the samples above were then stored at −20°C for further analysis. The small intestine including duodenum (from gizzard to pancreas-biliary ducts), jejunum (from pancreas-biliary ducts to Meckel’s diverticulum) and ileum (from Meckel’s diverticulum to ileocecal junction) were removed. Samples of the duodenum, jejunum, and ileum (1 cm cut from the midpoint) were stored in 10% neutral buffered formalin for 24 h for histomorphometrical study [26].

### Growth performance

The growth performance of broilers was evaluated by body weight gain (BWG), feed intake (FI) and feed conversion ratio (F/G). Body weights were recorded for each replicate at 1, 21 and 42 d of age. Before weighing at 21 and 42 d, feed was withdrawn for 12 h and water was provided *ad libitum*. Feed intake was also recorded during the 42-d of trial.

### Measurement of digestive enzyme activities of pancreas

The pancreas tissues were collected with following the method of Hu et al [27]. Briefly, pancreas tissue samples were homogenized (1:9, wt/vol) with ice-cold 0.86% physiological saline and then centrifuged at 4000 rpm at 4°C for 15 min. The supernatant was stored at −20°C for digestive enzyme activities. Amylase, protease and lipase enzyme activities were determined by using the commercial kits (Nanjing JianCheng Institute of Bioengineering, Jiangsu, China) according to the instructions of the manufacturer. Amylase and protease activities were expressed as units per milligram of protein, and lipase activity was expressed as units per gram of protein.

### Development of intestine and intestinal histomorphology

The length and weight of intestinal segments, the relative weight of intestinal segment to body weight and relative length of intestinal segment to body weight were calculated according to the study of Jin et al [28].

For histomorphometrical study, the intestine were later dehydrated in ethyl alcohols of increasing concentrations, cleared with xylene, and finally embedded in paraffin embedded wax. They were cut into 6 μm, placed on glass slides, and stained with haematoxylin and eosin. A total of ten slides for each intestine segment were prepared and analyzed using a light microscope for morphological observations. Images were collected using a Nikon ECLIPSE 80i light microscope equipped with a computer assisted morphometric system (Nikon Corporation, Tokyo, Japan). Villus height and crypt depth of twenty well oriented villi per image were measured using the Image-Pro Plus (IPP) software. Villus height was measured from the tip of the villi to the villus crypt junction, and crypt depth was defined from the valley between individual villus to the basal membrane. The villus height-to-crypt depth ratio (villus / crypt ratio) was also calculated [28–30].

### Antioxidant capacity of serum and intestinal mucosal

Nitric oxide (NO, μmol/L), total nitric oxide synthase (TNOS, U/L) and induce nitric oxide synthase (*i*NOS, U/L) were measured with commercial kits according to the manufacturer’s instructions (JianCheng Bioengineering Institute, Nanjing, China) [31, 32].

The mucosal of intestinal segments (jejunum and ileum) were scraped and then stored at −20°C for further analysis. After thawing, 0.2 to 0.3 g of mucosal scraping was homogenized with ice-cold physiological saline and centrifuged at 4000 rpm at 4°C for 10 min. Total protein content in mucosal scraping was determined according to the Bradford method. Antioxidant enzyme activities of catalase (CAT), superoxide dismutase (SOD), copper and zinc superoxide dismutase (CuZn-SOD), glutathione peroxidase (GSH-Px) and T-AOC were determined using corresponding diagnostic kits (Nanjing Jiancheng Bioengineering Institute, Nanjing, P. R. China) and expressed as unit per milligram of protein of intestine mucosal [33, 34].

### Statistical analysis

All data were analyzed by one-way analysis of variance (ANOVA) using SPSS statistical software (Ver.16.0 for windows, SPSS, Inc., Chicago, IL, USA). Differences among treatments were examined by Duncan’s multiple range test. Significance (*P*-value) was evaluated at 0.05. The means and standard errors (S.E.M) were also determined.

## Results

### Growth performance

The results showed that the SB group had significant increase in BWG and FI (P < 0.05) compared with CLI and CLI-B groups while there were no significant differences (P > 0.05) found in the F/G among the different dietary treatments at 21 d (Table 1). The supplementation of CLI-B decreased the F/G compared with the SB and CLI groups during 22–42 d of age. Additionally, a significant decreased (P < 0.05) in FI and F/G were found in broilers fed diets supplemented with CLI-B compared with the CON group. Furthermore, compared with the CLI group, CLI-B group significantly decreased F/G in broilers during the whole experimental period of 42 d.

**Table 1:** Effects of different treatments on growth performance of broilers chickens.

	Item^1^	CON ^2^	SB ^3^	CLI ^4^	CLI-B ^5^
**1–21 d**	BWG (kg/bird)	0.58 ± 0.01 ^ab^	0.64 ± 0.02 ^a^	0.56 ± 0.03 ^b^	0.56± 0.01 ^b^
	FI (kg/bird)	0.78 ± 0.01 ^ab^	0.81 ± 0.01 ^a^	0.74 ± 0.03 ^b^	0.73 ± 0.02 ^b^
	F/G (kg/kg)	1.35 ± 0.01	1.28± 0.03	1.33± 0.03	1.31± 0.02
**21–42 d**	BWG (kg/bird)	1.95 ± 0.05	1.84 ± 0.05	1.83 ± 0.03	1.84 ± 0.07
	FI (kg/bird)	3.48 ± 0.06	3.33 ± 0.13	3.46 ± 0.05	3.10 ± 0.021
	F/G (kg/kg)	1.79 ± 0.02 ^ab^	1.81 ± 0.04^a^	1.89 ± 0.01 ^a^	1.67 ± 0.07 ^b^
**1–42 d**	BWG (kg/bird)	2.52 ± 0.06	2.48 ± 0.06	2.39 ± 0.04	2.40 ± 0.06
	FI (kg/bird)	4.36 ± 0.07 ^a^	4.22 ± 0.11 ^ab^	4.30 ± 0.05 ^ab^	3.92 ± 0.20 ^b^
	F/G (kg/kg)	1.73 ± 0.02 ^a^	1.71 ± 0.03 ^ab^	1.80 ± 0.01 ^a^	1.63 ± 0.05 ^b^

*Note*.

^1^Abbreviations: BWG, body weight gain; FI, feed intake; F/G, feed conversion ratio.

^2^ Broilers fed a basal diet.

^3^ Broilers fed a basal diet supplemented with 0.05% sodium butyrate.

^4^ Broilers fed a basal diet supplemented with 1% clinoptilolite.

^5^ Broilers fed a basal diet supplemented 1% CLI-B.

^ab^ Means within a row with different letters (a, b) differ significantly (P < 0.05).

### Activities of pancreatic digestive enzymes

Pancreatic amylase activity was significantly increased (P < 0.05) in SB, CLI and CLI-B groups than the CON group in chickens at 21d. In chickens, lipases activity was significantly increased (P < 0.05) in the CLI-B group than CON group at both 21 d and 42 d of age. Moreover, the CLI-B supplementation significantly increased (P < 0.05) lipases activity than SB group at 21 d and CLI group at 42 d in chickens (Table 2).

**Table 2:** Effects of different treatments on digestive enzyme activities of pancreas of broilers chickens.

	Item	CON ^1^	SB ^2^	CLI ^3^	CLI-B ^4^
**21 d**	Amylase (U/mgprotein)	322.74 ± 36.19 ^b^	503.01 ± 30.53 ^a^	485.41 ± 35.33 ^a^	510.56 ± 16.13 ^a^
	Protease (U/gprotein)	5132.51±451.78	5889.78±198.02	6178.60±279.57	5776.39±472.17
	Lipase (U/mgprotein)	47.21 ± 4.71 ^b^	40.28 ±1.53 ^b^	52.33 ±5.88 ^ab^	63.19 ±3.96 ^a^
**42 d**	Amylase (U/mgprotein)	484.71 ±22.81	479.47±14.08	488.59±5.77	508.57±27.68
	Protease (U/gprotein)	4951.41±583.59	5872.49±506.08	5886.10±414.82	5473.76±334.87
	Lipase (U/mgprotein)	61.88 ±3.25 ^b^	65.62±4.74 ^b^	54.28±4.16 ^ab^	76.31±3.93 ^a^

*Note*.

^1^ Broilers fed a basal diet.

^2^ Broilers fed a basal diet supplemented with 0.05% sodium butyrate.

^3^ Broilers fed a basal diet supplemented with 1% clinoptilolite.

^4^ Broilers fed a basal diet supplemented 1% CLI-B.

^ab^ Means within a row with different letters (a, b) differ significantly (P < 0.05).

### Development of intestine

The results showed (Table 3) that CLI-B group had significantly increased (P < 0.05) the relative weight of duodenum than the CON group at 42 d. The relative length at 21 d and the relative weight of duodenum at 42 d in CLI-B group were significantly increased (P < 0.05) than SB group in chickens. The results showed (Table B in S1 File) that none of the experimental diets induced significant effects on the relative weight and length of jejunum at both 21 d and 42 d in chickens. Dietary CLI-B group had significant increased (P < 0.05) in the relative weight of ileum than the CON and SB groups at 21 d of age in chickens.

**Table 3:** Effects of different treatments on development of duodenum and ileum of broilers chickens.

	Item^1^	CON ^2^	SB ^3^	CLI ^4^	CLI-B ^5^
**Duodenum, 21 d**	Relative weight (g/kg BW)	8.75 ±0.31	8.39 ±0.32	8.69 ±0.28	9.24 ±0.39
	Relative length (cm/kg BW)	33.88 ±0.69 ^ab^	32.35 ±1.63 ^b^	35.25 ±1.31 ^ab^	37.04 ±1.43 ^a^
**Duodenum, 42 d**	Relative weight (g/kg BW)	4.01 ±0.15 ^b^	4.06 ±0.05 ^b^	4.25 ±0.09 ^ab^	4.49 ±0.16 ^a^
	Relative length (cm/kg BW)	9.95 ±0.37	9.77 ±0.32	10.59 ±0.25	10.61 ±0.39
**Ileum, 21 d**	Relative weight (g/kg BW)	10.49 ± 0.38 ^b^	10.10 ± 0.74 ^b^	11.53 ± 0.35 ^ab^	12.56 ± 0.90 ^a^
	Relative length (cm/kg BW)	59.87 ± 2.47	60.18 ± 3.79	60.80 ± 2.12	67.36 ± 3.14
**Ileum, 42 d**	Relative weight (g/kg BW)	8.45 ± 0.31	9.02 ± 0.35	9.01 ± 0.31	9.04 ± 0.26
	Relative length (cm/kg BW)	26.96 ± 1.70	28.02 ± 1.37	28.32 ± 1.27	29.46 ± 0.80

*Note*.

^1^Abbreviations: BW, body weight.

^2^ Broilers fed a basal diet.

^3^ Broilers fed a basal diet supplemented with 0.05% sodium butyrate.

^4^ Broilers fed a basal diet supplemented with 1% clinoptilolite.

^5^ Broilers fed a basal diet supplemented 1% CLI-B.

^ab^ Means within a row with different letters (a, b) differ significantly (P < 0.05).

### Intestinal histomorphology

**Fig 1:**
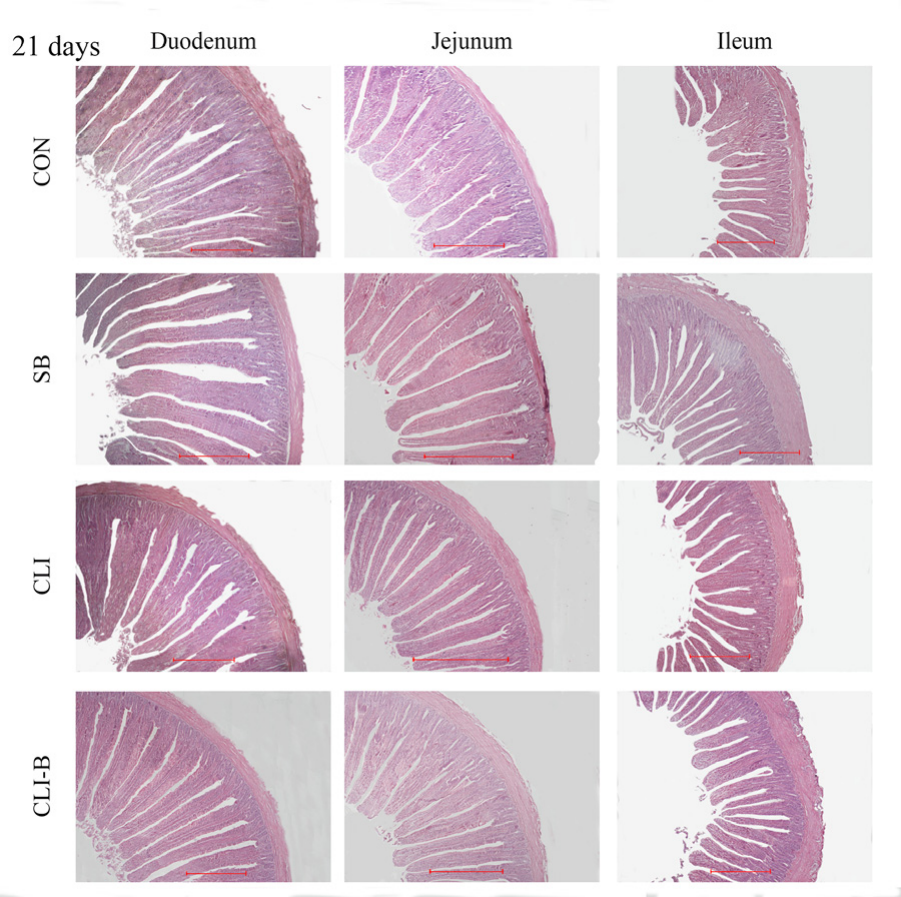
Haematoxylin and eosin (H&E) staining of duodenum, jejunum and ileum at 21 days of age. Note: Scale bar, 500 μm.

**Fig 2:**
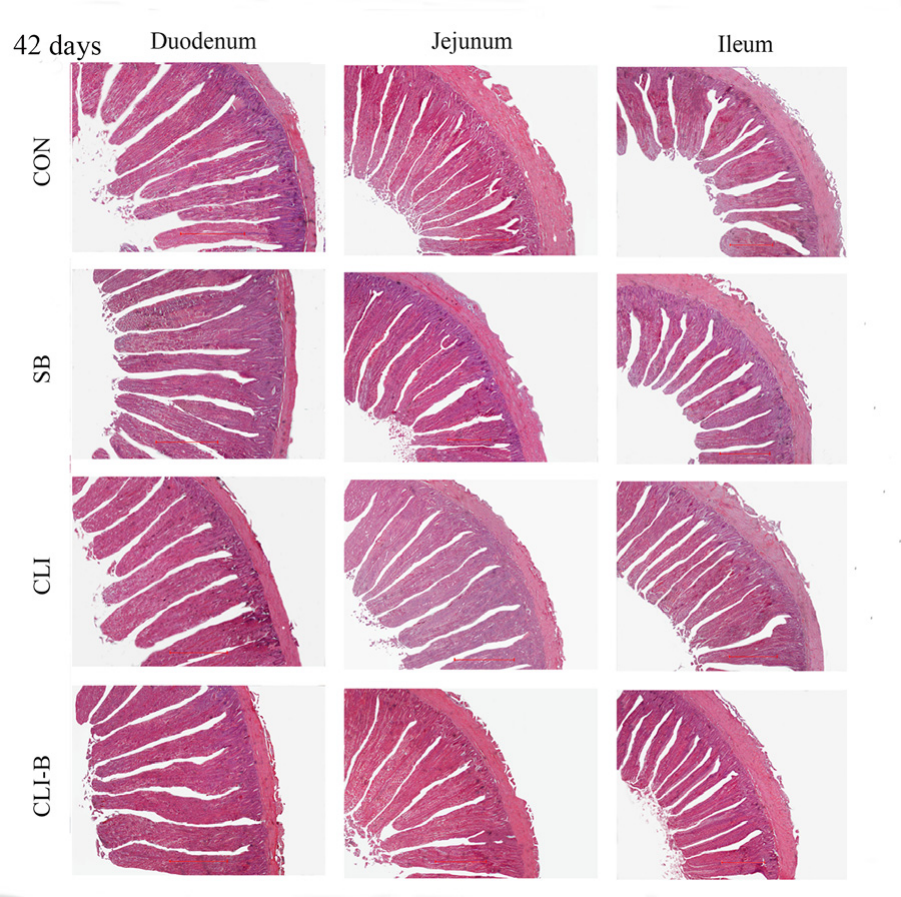
Haematoxylin and eosin (H&E) staining of duodenum, jejunum and ileum at 42 days of age. Note: Scale bar, 500 μm.

Haematoxylin and eosin (H&E) staining of duodenum, jejunum and ileum at 21 days and 42 days were presented in Fig 1 and Fig 2, respectively. No significant changes were found among all dietary groups in villus height of duodenum at both 21 d and 42 d (P > 0.05) of age, while a significant decreased in crypt depth and increased in villus/crypt ratio (P < 0.05) were found in the CLI-B group than other groups at 21 d in chickens (Table 4). In jejunum (Table 4), the villus height and villus/crypt ratio were significantly increased (P < 0.05) in SB and CLI-B groups than the CON group at 42 d. However, chickens in CLI-B group had significant increased (P < 0.05) in the villus height of jejunum than the CLI group at both 21 d and 42 d of age. Additionally, CLI-B group had significantly increased (P < 0.05) in villus/crypt ratio of jejunum than the CLI group at 42 d. Furthermore, compared with the CLI group, SB group had significantly increased (P < 0.05) in villus height of jejunum at 42 d. A significantly decreased in crypt depth and increased in villus/crypt ratio of ileum in chickens of SB and CLI-B groups (P < 0.05) were observed than the CON and CLI groups at 21 d of age. Supplementation of CLI-B in broiler chickens increased the villus/crypt ratio of ileum at 42 d compared with the CON group.

**Table 4:** Effects of different treatments on duodenum, jejunum and ileum histomorphology of broilers chickens.

	Item^1^	CON ^2^	SB ^3^	CLI ^4^	CLI-B ^5^
**Duodenum**	villus height	908.76±23.20	989.18±53.17	928.57±38.94	997.60±34.80
**21 d**	crypt depth	118.78±4.19 ^a^	113.22±2.96 ^a^	110.33±5.67 ^a^	93.25±6.57 ^b^
	villus/crypt ratio	7.70±0.36 ^b^	8.79±0.58 ^b^	8.45±0.26 ^b^	10.99±0.89 ^a^
**Duodenum**	villus height	1033.53±25.60	1126.18±47.59	1098.03±55.07	1190.83±66.95
**42 d**	crypt depth	103.64±5.52	99.93±6.75	95.78±5.61	94.78±5.28
	villus/crypt ratio	10.08 ±0.42	11.55±1.03	11.53±0.46	12.85±1.27
**Jejunum**	villus height	681.78 ±29.98 ^ab^	644.93±26.24 ^ab^	591.39±36.44 ^b^	719.03±40.32 ^a^
**21 d**	crypt depth	93.83±5.77	85.05±3.73	92.57±6.58	89.38±4.07
	villus/crypt ratio	7.38±0.50	7.67±0.51	6.48±0.46	8.13±0.59
**Jejunum**	villus height	848.40±34.93 ^b^	1108.28±43.02 ^a^	938.39±50.19 ^b^	1154.83±58.82 ^a^
**42 d**	crypt depth	104.73±3.48	94.48±7.09	92.71±5.23	89.08±4.89
	villus/crypt ratio	8.17±0.51 ^c^	11.97±0.80 ^ab^	10.23±0.63 ^b^^c^	13.17±1.00 ^a^
**Ileum**	villus height	494.93±19.72	573.93±52.48	569.87±28.21	575.53±31.11
**21 d**	crypt depth	89.83±2.96 ^a^	60.33±4.74 ^b^	83.67±5.13 ^a^	62.50±5.28 ^b^
	villus/crypt ratio	5.51±0.12 ^b^	9.61±0.63 ^a^	6.98±0.63 ^b^	9.56±0.95 ^a^
**Ileum**	villus height	783.08±30.01	845.65±34.87	862.05±24.78	864.34±32.38
**42 d**	crypt depth	115.31±3.76	102.45±4.78	109.05±7.02	100.69±2.50
	villus/crypt ratio	6.85±0.42 ^b^	8.37±0.57 ^ab^	8.09±0.58 ^ab^	8.65±0.52 ^a^

*Note*.

^1^Villus height, μm; crypt depth, μm; villus/crypt ratio, μm/μm.

^2^ Broilers fed a basal diet.

^3^ Broilers fed a basal diet supplemented with 0.05% sodium butyrate.

^4^ Broilers fed a basal diet supplemented with 1% clinoptilolite.

^5^ Broilers fed a basal diet supplemented 1% CLI-B.

^ab^ Means within a row with different letters (a, b) differ significantly (P < 0.05).

### Antioxidant capacity of serum and intestinal mucosal

As observed from Table 5, the serum NO content significantly decreased (P < 0.05) in chickens fed diets supplemented with butyrate than the CON group at both 21 d and 42 d of age. Broilers in CLI-B group had lower (P < 0.05) serum *i*NOS activity than the CON group at 21 d and 42 d. However, compared with SB and CLI groups, serum *i*NOS activity significantly decreased (P < 0.05) in the CLI-B group at 42 d.

**Table 5:** Effects of different treatments of CLI-B on serum NO content and NOS activity of broilers chickens.

	Item^1^	CON^2^	SB^3^	CLI ^4^	CLI-B ^5^
**21d**	NO (μmol/L)	13.32 ±0.37 ^a^	11.87 ±0.58 ^b^	11.72 ±0.43 ^b^	11.70 ±0.46 ^b^
	TNOS (U/mL)	9.20 ±0.11	8.80 ±0.23	8.81 ±0.23	8.50 ±0.35
	iNOS (U/mL)	7.30 ±0.22 ^a^	6.80 ±0.14 ^ab^	6.99 ±0.16 ^ab^	6.74 ±0.11 ^b^
**42d**	NO (μmol/L)	13.01 ±0.82 ^a^	10.23 ±0.26 ^b^	10.40 ±0.18 ^b^	10.50 ±0.37 ^b^
	TNOS (U/mL)	8.19 ±0.32	8.04 ±0.12	7.92 ±0.10	7.65 ±0.15
	iNOS (U/mL)	6.13 ±0.19 ^a^	6.06 ±0.14 ^a^	5.84 ±0.20 ^a^	5.13 ±0.22 ^b^

*Note*.

^1^Abbreviations: NO, nitric oxide; TNOS, total nitric oxide synthase; iNOS, induce nitric oxide synthase.

^2^ Broilers fed a basal diet.

^3^ Broilers fed a basal diet supplemented with 0.05% sodium butyrate.

^4^ Broilers fed a basal diet supplemented with 1% clinoptilolite.

^5^ Broilers fed a basal diet supplemented 1% CLI-B.

^ab^ Means within a row with different letters (a, b) differ significantly (P < 0.05).

Results of antioxidant capacity of the jejunum and ileum were shown in Table 6. Chickens in CLI-B group had significant increased (P < 0.05) in the CAT enzyme activity at 21 d of age, GSH-Px enzyme activity at 42 d of age and T-AOC in the mucosa of jejunum at both 21 d and 42 d of age than the CON group at the same period. Compared with the SB group, the T-AOC at 21 d of age, while, both SOD and GSH-Px enzyme activities at 42 d of age were significantly increased (P < 0.05) in the mucosa of jejunum of CLI-B group in chickens. Supplementation of CLI-B in chickens significantly increased (P < 0.05) the T-AOC and CAT enzyme activity at 21 d of age, while, GSH-Px enzyme activity at 42 d of age in the mucosa of jejunum compared with CLI group. Furthermore, CLI group had significantly increased (P < 0.05) the SOD enzyme activity in the mucosa of jejunum than the SB group at 42 d in chickens. CLI-B supplementation with diets significantly increased (P < 0.05) the CuZn-SOD enzyme activity at 21 d and 42 d, while, activities of SOD and GSH-Px enzymes and T-AOC at 42 d of age in the mucosa of ileum than the CON and SB groups at the same period in chickens. Compared with CLI group, CLI-B group significantly increased (P < 0.05) the activities of CuZn-SOD (at 21 d and 42 d) and GSH-Px enzyme (at 42 d) of age in chickens. Moreover, CLI group significantly increased (P < 0.05) the activities of SOD and GSH-Px enzymes and the T-AOC in the mucosa of ileum than the CON and SB groups at 42 d of age in chickens.

**Table 6:** Effects of different treatments of CLI-B on antioxidant capacity of jejunum mucosal of broilers chickens.

	*Item*^1^	*CON*^2^	*SB*^3^	*CLI* ^4^	*CLI-B* ^5^
***Jejunum*, *21 d***	*CAT (U/mg protein)*	*5*.*89±0*.*36*^b^	*6*.*06±0*.*51*^ab^	*5*.*95±0*.*33* ^b^	*7*.*25±0*.*45*^a^
	*SOD (U/mg protein)*	*89*.*95±2*.*25*	*92*.*61 ±3*.*41*	*93*.*62 ±3*.*06*	*105*.*96 ±10*.*65*
	*CuZn-SOD (U/mg protein)*	*59*.*79 ±3*.*03*	*51*.*87 ±3*.*78*	*51*.*81 ±2*.*79*	*59*.*80 ±5*.*72*
	*GSH-Px (U/mg protein)*	*23*.*50 ±2*.*01*	*27*.*70 ±2*.*68*	*23*.*11 ±0*.*83*	*28*.*05 ±1*.*52*
	*T-AOC (U/mg protein)*	*0*.*26 ±0*.*02* ^b^	*0*.*28 ±0*.*03* ^b^	*0*.*26 ±0*.*02* ^b^	*0*.*36 ±0*.*02*^a^
***Jejunum*, *42 d***	*CAT (U/mg protein)*	*3*.*47 ±0*.*27*	*3*.*58 ±0*.*11*	*3*.*45 ±0*.*20*	*3*.*93 ±0*.*33*
	*SOD (U/mg protein)*	*100*.*82 ±4*.*80* ^ab^	*93*.*50 ±6*.*31* ^b^	*111*.*28±3*.*99* ^a^	*110*.*19 ±5*.*34* ^a^
	*CuZn-SOD (U/mg protein)*	*71*.*71 ±5*.*60*	*74*.*41 ±4*.*04*	*76*.*54 ±3*.*57*	*84*.*07 ±3*.*92*
	*GSH-Px (U/mg protein)*	*38*.*24 ±5*.*06* ^b^	*38*.*98 ±1*.*49* ^b^	*44*.*89±5*.*79* ^b^	*70*.*00 ±5*.*62* ^a^
	*T-AOC (U/mg protein)*	*0*.*10 ±0*.*01* ^b^	*0*.*13 ±0*.*02* ^ab^	*0*.*11 ±0*.*02* ^ab^	*0*.*15 ±0*.*01* ^a^
***Ileum*, *21 d***	*CAT (U/mg protein)*	*4*.*19 ± 0*.*25*	*4*.*38 ±0*.*42*	*4*.*41 ±0*.*43*	*4*.*99 ±0*.*39*
	*SOD (U/mg protein)*	*67*.*91 ± 3*.*67*	*71*.*57 ±6*.*82*	*68*.*38 ±3*.*68*	*78*.*74 ±6*.*40*
	*CuZn-SOD (U/mg protein)*	*52*.*55 ±4*.*12* ^b^	*47*.*89 ±3*.*02* ^b^	*47*.*00 ±2*.*55* ^b^	*92*.*59 ±9*.*22* ^a^
	*GSH-Px (U/mg protein)*	*50*.*27 ±2*.*60*	*48*.*83 ±3*.*80*	*52*.*82 ±3*.*29*	*55*.*98 ±4*.*10*
	*T-AOC (U/mg protein)*	*0*.*22 ±0*.*02*	*0*.*26 ±0*.*02*	*0*.*25 ±0*.*02*	*0*.*27 ±0*.*03*
***Ileum*, *42 d***	*CAT (U/mg protein)*	*6*.*71 ±0*.*42*	*7*.*11 ±0*.*23*	*7*.*18 ±0*.*38*	*7*.*37 ±0*.*60*
	*SOD (U/mg protein)*	*82*.*88 ±7*.*62* ^b^	*84*.*28 ±4*.*82* ^b^	*115*.*49 ±9*.*73* ^a^	*127*.*10 ±6*.*98* ^a^
	*CuZn-SOD (U/mg protein)*	*50*.*52 ±3*.*00* ^b^	*53*.*42 ±4*.*11* ^b^	*63*.*38 ±7*.*31* ^b^	*82*.*11 ±7*.*59* ^a^
	*GSH-Px (U/mg protein)*	*54*.*12 ±5*.*08* ^*c*^	*54*.*57 ±3*.*19* ^*c*^	*72*.*15 ±3*.*76* ^b^	*113*.*32 ±4*.*70* ^a^
	*T-AOC (U/mg protein)*	*0*.*17 ±0*.*02* ^b^	*0*.*19 ±0*.*01* ^b^	*0*.*24 ±0*.*01* ^a^	*0*.*27 ±0*.*02* ^a^

*Note*.

^1^Abbreviations: CAT, catalase; SOD, superoxide dismutase; CuZn-SOD, copper and zinc superoxide dismutase; GSH-Px, glutathione peroxidase; T-AOC, total antioxidant capacity.

^2^ Broilers fed a basal diet.

^3^ Broilers fed a basal diet supplemented with 0.05% sodium butyrate.

^4^ Broilers fed a basal diet supplemented with 1% clinoptilolite.

^5^ Broilers fed a basal diet supplemented 1% CLI-B.

^ab^ Means within a row with different letters (a, b) differ significantly (P < 0.05).

## Discussion

### Growth performance

Dietary supplementation of sodium butyrate could increase the average body weight gain and feed consumption while reducing the F/G [35]. Previous studies also demonstrated that dietary sodium butyrate had a positive effect on body weight gain when supplemented at levels of 500 and 2000 mg/kg from 0 to 21 days, but a negative effect on the F/G with a supplemented level over 2000 mg/kg. Too high or too low levels of sodium butyrate had negative effects on the F/G [36]. Therefore, a diet supplemented with 500 mg/kg sodium butyrate was chosen as a control in our experiment due to its positive effect on body weight gain [4]. Although, there were no significantly differences in the F/G between SB and CON groups, however, SB group significantly increased body weight gain and feed intake compared with the other treatments at 21 d of age in chickens (Table 1). Different effects of clinoptilolite on growth performance have been reported in chickens. Olver found that the addition of clinoptilolite in layer diets increased feed intake in laying hens [37], while others found that there was no significant effect on feed intake by the addition of clinoptilolite into the diets of laying hens [38]. However, another study concluded that a layer diets supplemented with the sodium clinoptilolite at 1.5% level had an adverse effect on feed intake in layer hens [39]. Most of studies reported there was no significant improvement on growth performance when supplementation of clinoptilolite into the diet, while there were very few studies showed adverse effects [40]. In our present study, no significant difference was found on growth performance between CLI and control group, which showed that addition of CLI in the diets did no harm to the growth performance of broiler chickens. Moreover, CLI-B supplementation had decreased the feed intake compared with the CON group during the whole period but also decreased F/G at 42 d and the whole period which indicated that the addition of CLI-B in diets could increase the feed conversion efficiency in broilers. The differences between CLI and CLI-B groups may be caused by the butyrate loaded into the clinoptilolite [35], which is an evidence that butyrate was successfully loaded into the clinoptilolite. The results also showed that the addition of CLI-B could improve the feed conversion efficiency than the SB or CLI groups at 42 d or even the whole period.

### Digestive enzymes of pancreas assay

The pancreas secretes a series of enzymes that are essential for the digestion of nutrients. Thus, the enzyme activities in pancreas could reflect the level of endogenous enzymes which are synthesized and locally stored [41]. Previous studies showed that the sodium butyrate supplementation modified some digestive enzyme activities in the calves serving as a basis for enhanced digestibility and absorption [42] as well as microstructure of the small intestine in piglets [43]; Supplementation with clinoptilolite significantly improved the activities of digestive enzymes in the small intestinal contents [44]. Based on the results of this study, the CLI-B supplementation in broiler chickens had higher pancreatic enzyme activities especially amylase and lipase than other experimental groups which indicated CLI-B could improve the digestibility and utilization of feed. Improved activities of pancreatic digestive enzymes may explain the decreased of F/G in CLI-B group than other groups in chickens. The positive effect of CLI-B on broilers may be an attribute to the interaction of clinoptilolite and butyrate; the clinoptilolite, serving as a controlled-release carrier, might have modified butyrate release which improved nutrient utilization and enzyme activity, therefore benefit the feed efficiency in broilers chickens [29].

### Development of intestine

The intestine is a fast turnover tissue of the whole gastrointestinal tract in the living body. The improvement of gut health is essential for the poultry welfare as well as the productivity. Any kind of gut damage will result in poor gut health, which will in turn, decrease nutrient utilization efficiency [45]. In our study, the increased relative weight and length of intestine indicated that supplementation of CLI-B help improve the development of intestine thus improve the health of gut. The benefits of CLI-B on development of intestine may be associated with slower passage of nutrient through the digestive tract with the addition of clinoptilolite as well as the benefits of butyrate. The higher values in CLI-B group than the SB and CON groups also suggested that the combination of clinoptilolite and butyrate have potential application and better effects than supplementation butyrate individually.

### Intestinal histomorphology

The structure of the intestinal mucosal reflects gut health status. Longer villi can increase intestinal surface area thus support better nutrient absorption which is also an indication of healthy development of intestine in response to the use of feed additives [46]. A large crypt can indicate greater tissue turnover and higher demand for new tissues [47]. In present study, supplementation of sodium butyrate showed beneficial effects for intestinal structure and morphology. Moreover, lower crypt depth and higher villus/crypt ratio indicated the addition of CLI-B increased the absorption of nutrients, decreased the secretion of the gut, improved the resistance of disease and increased the overall performance [47, 48]. Both the sustained release of butyrate and the damage protection capability of clinoptilolite in the mucosal layers provided beneficial supports to maintain intact mucosal structure[24, 29]. A tendency toward increased villus height throughout the intestinal segments of broilers fed with natural zeolite including plant extract was observed [49], which demonstrated the positive effect of feed additive using zeolite clinoptilolite as a carrier. In short, the addition of CLI-B increased nutrient absorption, improved intestine health than addition of clinoptilolite and sodium butyrate alone.

### Serum NO content and NOS enzyme activity

Nitric oxide (NO) is an important signaling molecule involved in various developmental, pathological and physiological processes generated in neutrophils, certain T and B cell lines [50]. The expression of *i*NOS (a rate-limiting enzyme synthesis NO) are generally recognized to reflect the antioxidant capacity of animals like antioxidant enzymes activities. NO modulates the production of cytokines, chemokines and growth factors [51]. Higher level of NO produced by *i*NOS leads to potent antimicrobial effects to control infections and hence is critical in immune defense [51] and the occurrence of apoptosis and/or necrosis in immune cells [52, 53]. The current study found that three treatments (SB, CLI and CLI-B) significantly decreased the content of NO (P < 0.05) than the CON group at 21 d and 42 d. Natural clinoptilolite have positive influence on the inflammatory processes by decreasing the synthesis of nitric oxide and superoxide anions [17]. Both Kanika et al and our study reported that sodium butyrate significantly decreased the expression of *i*NOS [54]. The decreased *i*NOS in CLI-B group may attribute to the adsorption of butyrate into clinoptilolite and also might be due to a sustained slow release of butyrate from CLI-B in the gastrointestinal tract. The decreased levels of NO and *i*NOS in CLI-B group indicated that the supplementation of CLI-B could improve antioxidant capacity in broilers which further showed that the addition of SB, CLI and CLI-B did no harm the health of broilers.

### Intestinal mucosal antioxidant capacity

Antioxidant enzymes are indispensable key factors against oxidative stress induced by xenobiotic in animal defense system [55]. The antioxidant enzyme defense system consists of CAT, SOD, GSH-Px. SOD dismutase superoxide radicals (HO^.^_2_-/O^.^_2_-) to the less toxic H_2_O_2_ while CAT and GSH-Px detoxify H_2_O_2_ into O_2_ and H_2_O [56]. The T-AOC includes a number of antioxidant enzymes and related biomolecules with the ability to remove free radicals of a specific organ or a living organism. The level of T-AOC reflects the total antioxidant ability [56].

Some *in vitro* studies indicated that butyrate could increase the activity of antioxidant enzymes. The activity of antioxidant enzymes in normal colon cells significantly increased after exposure to butyrate environment [57]. Orchel *et al*. also demonstrated the total SOD activity increased in Caco-2 colon carcinoma cells after 4 days of butyrate treatment [58]. In this experiment, although addition of SB did not significantly increase the antioxidant enzymes activity, it showed higher values of antioxidant enzyme activities than the CON group. Previous studies indicated that addition of clinoptilolite into the diet of broiler chickens could significantly increase the activity of antioxidant enzymes [59] which was also showed in this study. In the present study, it indicated that higher oxidative susceptibility in CLI-B group than other groups of jejunum and ileum at either 21 d or 42 d. The antioxidant properties of feed additives may also act within the digestive tract and improve overall gut functions [60]. The changes in antioxidant properties observed in this study indicated that the CLI-B contributed to improve the antioxidant properties than adding sodium butyrate and clinoptilolite alone. The antioxidant capacity may help improve the development of the digestive tract and are indications of gut health effects of CLI-B.

## Conclusions

The results of this study showed butyrate was successfully loaded into the clinoptilolite. Dietary supplementation of CLI-B decreased the F/G, increased activities of digestive enzymes, promoted the development and health of intestine by increasing the relative weight and length of intestine, decreasing the crypt depth as well as increasing the villus height and villus / crypt ratio. Furthermore, the CLI-B could improve the antioxidant capacity of intestine by decreasing the NO content, *i*NOS activity, and increasing the antioxidant enzyme activities in chickens. In addition, the CLI-B supplementation had better improvement in the development of intestine and antioxidant statuses of broiler chickens than supplementation with clinoptilolite or butyrate sodium alone. In conclusion, 1% butyrate loaded clinoptilolite supplementation is a practical management application with economic benefits for the broiler industry.

## Supporting Information

S1 File **Table A. Ingredients and nutrient composition of broiler diets, as-fed basis.** The starter basal diet from 1 d to 21 d of age and a grower basal diet from 22 d to 42 d of age for broiler chickens. **Table B. Effects of different treatments on development of jejunum of broilers chickens.** The relative weight and length of jejunum in different treatment groups at both 21 d and 42 d of age.
(DOCX)

## Abbreviations

CLI: clinoptilolite
CLI-B: butyrate loaded clinoptilolite
SB: sodium butyrate
DTA: differential thermal analysis
BWG: body weight gain
FI: feed intake
F/G: feed conversion ratio
NO: nitric oxide
TNOS: total nitric oxide synthase
*i*NOS: induce nitric oxide synthase
CAT: catalase
SOD: superoxide dismutase
CuZn-SOD: copper and zinc superoxide dismutase
GSH-Px: glutathione peroxidase
T-AOC: total antioxidant capacity

## References

[1] Wächtershäuser A, Stein J. Rationale for the luminal provision of butyrate in intestinal diseases. Eur J Nutr. 2000;39(4):164–71. 10.1007/s003940070020 11079736

[2] Scheppach W, Bartram P, Richter A, Richter F, Liepold H, Dusel G, et al Effect of short-chain fatty acids on the human colonic mucosa in vitro. Journal of Parenteral and Enteral Nutrition. 1992;16(1):43–8. 10.1177/014860719201600143 1738218

[3] Ricke S. Perspectives on the use of organic acids and short chain fatty acids as antimicrobials. Poultry Science. 2003;82(4):632–9. 10.1093/ps/82.4.632 12710485

[4] Hu Z, Guo Y. Effects of dietary sodium butyrate supplementation on the intestinal morphological structure, absorptive function and gut flora in chickens. Animal Feed Science and Technology. 2007;132(3–4):240–9. 10.1016/j.anifeedsci.2006.03.017.

[5] Xiao M, Liu YG, Zou MC, Zou F. Sodium butyrate induces apoptosis of human colon cancer cells by modulating ERK and Sphingosine Kinase 2. Biomedical and Environmental Sciences. 2014;27(3):197–203. 10.3967/bes2014.040 24709100

[6] Gao Z, Yin J, Zhang J, Ward RE, Martin RJ, Lefevre M, et al Butyrate improves insulin sensitivity and increases energy expenditure in mice. Diabetes. 2009;58(7):1509–17. Epub 2009/04/16. 10.2337/db08-1637 ; PubMed Central PMCID: PMCPmc2699871. 19366864 PMC2699871

[7] Matis G, Kulcsar A, Turowski V, Febel H, Neogrady Z, Huber K. Effects of oral butyrate application on insulin signaling in various tissues of chickens. Domest Anim Endocrinol. 2015;50:26–31. Epub 2014/09/23. 10.1016/j.domaniend.2014.07.004 . 25240231

[8] Yoo CB, Jones PA. Epigenetic therapy of cancer: past, present and future. Nature reviews Drug discovery. 2006;5(1):37–50. Epub 2006/02/18. 10.1038/nrd1930 . 16485345

[9] Hume ME, Corrier DE, Ivie GW, Deloach JR. Metabolism of [14C]propionic acid in broiler chicks. Poult Sci. 1993;72(5):786–93. Epub 1993/05/01. . 8502603 10.3382/ps.0720786

[10] Thompson JL, Hinton M. Antibacterial activity of formic and propionic acids in the diet of hens on *Salmonellas* in the crop. British poultry science. 1997;38(1):59–65. Epub 1997/03/01. 10.1080/00071669708417941 . 9088614

[11] Nezamzadeh-Ejhieh A, Raja G. Modification of nanoclinoptilolite zeolite with hexadecyltrimethylammonium surfactant as an active ingredient of chromate-selective membrane electrode. Journal of Chemistry. 2012;2013.

[12] Armbruster T. Clinoptilotite-heulandite: applications and basic research. Studies in surface science and catalysis. 2001;135:13–27. 10.1016/S0167-2991(01)81183-6

[13] Papaioannou D, Katsoulos P, Panousis N, Karatzias H. The role of natural and synthetic zeolites as feed additives on the prevention and/or the treatment of certain farm animal diseases: a review. Microporous and mesoporous materials. 2005;84(1):161–70. 10.1016/j.micromeso.2005.05.030 PMC7106472 32288627

[14] Oguz H. A review from experimental trials on detoxification of aflatoxin in poultry feed. Eurasian J Vet Sci. 2011;27(1):1–12.

[15] Pavelic K, Katic M, Sverko V, Marotti T, Bosnjak B, Balog T, et al Immunostimulatory effect of natural clinoptilolite as a possible mechanism of its antimetastatic ability. Journal of cancer research and clinical oncology. 2002;128(1):37–44. 10.1007/s00432-001-0301-6 11862470 PMC12164407

[16] Jung B, Toan N, Cho S, Ko J, Jung Y, Lee B. Dietary aluminosilicate supplement enhances immune activity in mice and reinforces clearance of porcine circovirus type 2 in experimentally infected pigs. Veterinary microbiology. 2010;143(2):117–25. 10.1016/j.vetmic.2009.11.009 20022715

[17] Šverko V, Sobočanec S, Balog T, Colić M, Marotti T. Natural micronised clinoptilolite and clinoptilolite mixtures with Urtica dioica L. extract as possible antioxidants. Food Technology and Biotechnology. 2004;42(3):189–92.

[18] Zarcovic N, Zarcovic K, Kralj M, Borovic S, Sabolovic S, Blazi MP, et al Anticancer and antioxidative effects of micronized zeolite clinoptilolite. Anticancer research. 2003;23(2):1589–96. 12820427

[19] Vilaça N, Amorim R, Machado AF, Parpot P, Pereira MF, Sardo M, et al Potentiation of 5-fluorouracil encapsulated in zeolites as drug delivery systems for in vitro models of colorectal carcinoma. Colloids & Surfaces B Biointerfaces. 2013;112(3):237–44. 10.1016/j.colsurfb.2013.07.042 23988779

[20] Spanakis M, Bouropoulos N, Theodoropoulos D, Sygellou L, Ewart S, Moschovi AM, et al Controlled release of 5-fluorouracil from microporous zeolites. Nanomedicine Nanotechnology Biology & Medicine. 2014;10(1):197–205. Controlled release of 5-fluorouracil from microporous zeolites. 10.1016/j.nano.2013.06.016 23916887

[21] Jevtić S, Grujić S, Hrenović J, Rajić N. Surfactant-modified clinoptilolite as a salicylate carrier, salicylate kinetic release and its antibacterial activity. Microporous and Mesoporous Materials. 2012;159(0):30–5. 10.1016/j.micromeso.2012.04.014.

[22] Garcia-Basabe Y, Rodriguez-Iznaga I, de Menorval L-C, Llewellyn P, Maurin G, Lewis DW, et al Step-wise dealumination of natural clinoptilolite: Structural and physicochemical characterization. Microporous and Mesoporous Materials. 2010;135(1–3):187–96. 10.1016/j.micromeso.2010.07.008.

[23] Rožić M, Cerjan-Stefanović Š, Kurajica S, Maěefat MR, Margeta K, Farkaš A. Decationization and dealumination of clinoptilolite tuff and ammonium exchange on acid-modified tuff. Journal of Colloid and Interface Science. 2005;284(1):48–56. 10.1016/j.jcis.2004.09.061. 15752783

[24] Wu QJ, Zhou YM, Wu YN, Zhang LL, Wang T. The effects of natural and modified clinoptilolite on intestinal barrier function and immune response to LPS in broiler chickens. Veterinary immunology and immunopathology. 2013;153(1):70–6. 10.1016/j.vetimm.2013.02.006 23453767

[25] Carraro A, De Giacomo A, Giannossi ML, Medici L, Muscarella M, Palazzo L, et al Clay minerals as adsorbents of aflatoxin M1 from contaminated milk and effects on milk quality. Applied Clay Science. 2014;88–89:92–9. 10.1016/j.clay.2013.11.028.

[26] Oliveira L, Madrid J, Ramis G, Martínez S, Orengo J, Villodre C, et al Adding crude glycerin to nursery pig diet: Effect on nutrient digestibility, metabolic status, intestinal morphology and intestinal cytokine expression. Livestock Science. 2014;167(0):227–35. 10.1016/j.livsci.2014.05.013.

[27] Hu C, Song J, You Z, Luan Z, Li W. Zinc oxide-montmorillonite hybrid influences diarrhea, intestinal mucosal integrity, and digestive enzyme activity in weaned pigs. Biol Trace Elem Res. 2012;149(2):190–6. Epub 2012/04/28. 10.1007/s12011-012-9422-9 . 22539019

[28] Jin L, Gao Y-y, Ye H, Wang W-c, Lin Z-p, Yang H-y, et al Effects of dietary fiber and grit on performance, gastrointestinal tract development, lipometabolism, and grit retention of goslings. Journal of Integrative Agriculture. 2014;13(12):2731–40. 10.1016/S2095-3119(13)60729-7.

[29] Tang ZG, Wen C, Wang LC, Wang T, Zhou YM. Effects of zinc-bearing clinoptilolite on growth performance, cecal microflora and intestinal mucosal function of broiler chickens. Animal Feed Science and Technology. 2014;189(0):98–106. 10.1016/j.anifeedsci.2013.12.014.

[30] Dong L, Zhong X, Ahmad H, Li W, Wang Y, Zhang L, et al Intrauterine growth restriction impairs small intestinal mucosal immunity in neonatal piglets. Journal of Histochemistry & Cytochemistry. 2014:1–9. 10.1369/0022155414532655 PMC4174621 24710659

[31] Li Z, Liu J, Liu S, Li X, Yi D, Zhao M. Improvement of vascular function by acute and chronic treatment with the GPR30 agonist G1 in experimental diabetes mellitus. PloS one. 2012;7(6):e38787 10.1371/journal.pone.0038787 22679517 PMC3367949

[32] Qu Z, Miao W, Hu S, Li C, Zhuo X, Zong Y, et al N-methyl-D-aspartate receptor-dependent denitrosylation of neuronal nitric oxide synthase increase the enzyme activity. PloS one. 2012;7(12). 10.1371/journal.pone.0052788 PMC3532120 23285183

[33] Cao M, Che L, Wang J, Yang M, Su G, Fang Z, et al Effects of maternal over- and undernutrition on intestinal morphology, enzyme activity, and gene expression of nutrient transporters in newborn and weaned pigs. Nutrition. 2014;30(11–12):1442–7. 10.1016/j.nut.2014.04.016 25280425

[34] Wang O, Liu J, Cheng Q, Guo X, Wang Y, Zhao L, et al Effects of Ferulic Acid and γ-Oryzanol on High-Fat and High-Fructose Diet-Induced Metabolic Syndrome in Rats. PloS one. 2015;10(2). 10.1371/journal.pone.0118135 PMC4315454 25646799

[35] Galfi P, Bokori J. Feeding trial in pigs with a diet containing sodium n-butyrate. Acta veterinaria Hungarica. 1990;38(1–2):3–17. Epub 1990/01/01. . 2100936

[36] Furuse M, Yang S, Niwa N, Okumura J. Effect of short chain fatty acids on the performance and intestinal weight in germ‐free and conventional chicks. British poultry science. 1991;32(1):159–65. 10.1080/00071669108417337 2049620

[37] Olver M. Effect of feeding clinoptilolite (zeolite) to three strains of laying hens. British poultry science. 1989;30(1):115–21. 10.1080/00071668908417130 2545313

[38] Roland D, Rabon H, Frost T, Laurent S, Barnes D. Response of commercial Leghorns to sodium aluminosilicate when fed different levels and sources of available phosphorus. Poultry science. 1990;69(12):2157–64. 10.3382/ps.0692157 1964736

[39] Miles R, Harms R, Laurent S. Influence of sodium zeolite A (Ethacal) on laying hen performance. Nutrition reports international. 1986;34(6):1097–103.

[40] Evans M. Zeolites: do they have a role in poultry production. 1989.

[41] Wen C, Wang LC, Zhou YM, Jiang ZY, Wang T. Effect of enzyme preparation on egg production, nutrient retention, digestive enzyme activities and pancreatic enzyme messenger RNA expression of late-phase laying hens. Animal Feed Science & Technology. 2012;172(3–4):180–6. 10.1016/j.anifeedsci.2011.11.012

[42] Guilloteau P, Zabielski R, David J, Blum J, Morisset J, Biernat M, et al Sodium-butyrate as a growth promoter in milk replacer formula for young calves. Journal of dairy science. 2009;92(3):1038–49. 10.3168/jds.2008-1213 19233797

[43] Kotunia A, Wolinski J, Laubitz D, Jurkowska M, Rome V, Guilloteau P, et al Effect of sodium butyrate on the small intestine. Journal of Physiology and Pharmacology. 2004;55:59–68. 15608361

[44] Wu QJ, Zhou YM, Wu YN, Wang T. Intestinal Development and Function of Broiler Chickens on Diets Supplemented with Clinoptilolite. Asian-Australasian Journal of Animal Sciences. 2013;26(7):987–94. 10.5713/ajas.2012.12545 . 25049877 PMC4093499

[45] Choct M. Managing gut health through nutrition. British poultry science. 2009;50(1):9–15. Epub 2009/02/24. 10.1080/00071660802538632 . 19234925

[46] Olukosi OA, Dono ND. Modification of digesta pH and intestinal morphology with the use of benzoic acid or phytobiotics and the effects on broiler chicken growth performance and energy and nutrient utilization. Journal of animal science. 2014;92(9):3945–53. 10.2527/jas.2013-6368 25085400

[47] Hu CH, Gu LY, Luan ZS, Song J, Zhu K. Effects of montmorillonite–zinc oxide hybrid on performance, diarrhea, intestinal permeability and morphology of weanling pigs. Animal Feed Science & Technology. 2012;177(177):108–15. 10.1016/j.anifeedsci.2012.07.028

[48] Nabuurs MJ, Hoogendoorn A, Ej VDM, van Osta AL. Villus height and crypt depth in weaned and unweaned pigs, reared under various circumstances in The Netherlands. Research in Veterinary Science. 1993;55(1):78–84. 10.1016/0034-5288(93)90038-H 8378616

[49] Incharoen T, Khambualai O, Yamauchi K. Morphological assessment of the small intestine of broilers fed dietary natural zeolite including plant extract. Journal of Agricultural Science and Technology A. 2011:1284–7.

[50] Koide N, Sugiyama T, Mu MM, Mori I, Yoshida T, Hamano T, et al Gamma interferon-induced nitric oxide production in mouse CD5^+^ B1-Like cell line and its association with apoptotic cell death. Microbiology and immunology. 2003;47(9):669–79. Epub 2003/10/31. . 14584614 10.1111/j.1348-0421.2003.tb03430.x

[51] Bogdan C. Nitric oxide and the immune response. Nature immunology. 2001;2(10):907–16. Epub 2001/09/29. 10.1038/ni1001-907 . 11577346

[52] Karpuzoglu E, Ahmed SA. Estrogen regulation of nitric oxide and inducible nitric oxide synthase (*i*NOS) in immune cells: Implications for immunity, autoimmune diseases, and apoptosis. Nitric Oxide. 2006;15(3):177–86. 10.1016/j.niox.2006.03.009. 16647869

[53] Messmer UK, Lapetina EG, Brune B. Nitric oxide-induced apoptosis in RAW 264.7 macrophages is antagonized by protein kinase C- and protein kinase A-activating compounds. Molecular pharmacology. 1995;47(4):757–65. Epub 1995/04/01. . 7723736

[54] Kanika G, Khan S, Jena G. Sodium butyrate ameliorates L‐arginine‐induced pancreatitis and associated fibrosis in wistar rat: role of inflammation and nitrosative stress. Journal of biochemical and molecular toxicology. 2015;29(8):349–59. 10.1002/jbt.21698 25774002

[55] Hwang PP, Tsai YN. Effects of arsenic on osmoregulation in the tilapia Oreochromis mossambicus reared in seawater. Marine Biology. 1993;117(4):551–8. 10.1007/BF00349765

[56] Ahmad H, Tian J, Wang J, Khan MA, Wang Y, Zhang L, et al Effects of dietary sodium selenite and selenium yeast on antioxidant enzyme activities and oxidative stability of chicken breast meat. Journal of agricultural and food chemistry. 2012;60(29):7111–20. 10.1021/jf3017207 22732007

[57] Jahns F, Wilhelm A, Jablonowski N, Mothes H, Greulich KO, Glei M. Butyrate modulates antioxidant enzyme expression in malignant and non‐malignant human colon tissues. Molecular carcinogenesis. 2015;54(4):249–60. 10.1002/mc.22102 24677319

[58] Orchel A, Gruchlik A, Weglarz L, Dzierzewicz Z. Influence of sodium butyrate on antioxidative enzymes activity in Caco-2 cell lines. Acta poloniae pharmaceutica. 2006;63(5):441–2. Epub 2007/03/16. . 17357609

[59] Wu Y, Wu Q, Zhou Y, Ahmad H, Wang T. Effects of clinoptilolite on growth performance and antioxidant status in broilers. Biol Trace Elem Res. 2013;155(2):228–35. 10.1007/s12011-013-9777-6 23949793 PMC3785709

[60] Halliwell B, Zhao K, Whiteman M. The gastrointestinal tract: a major site of antioxidant action? Free radical research. 2000;33(6):819–30. 10.1080/10715760000301341 11237104

