# Supplementary material for: Influence of Butyrate Loaded Clinoptilolite Dietary Supplementation on Growth Performance, Development of Intestine and Antioxidant Capacity in Broiler Chickens
Source: PLoS One. 2016 Apr 22;11(4):e0154410. doi: 10.1371/journal.pone.0154410 (PMC4841535; doi:10.1371/journal.pone.0154410)
Supplement: S1 File — Table A. Ingredients and nutrient composition of broiler diets, as-fed basis. The starter basal diet from 1 d to 21 d of age and a grower basal diet from 22 d to 42 d of age for broiler chickens. Table B. Effects of different treatments on development of jejunum of broilers chickens. The relative weight and length of jejunum in different treatment groups at both 21 d and 42 d of age. (DOCX) [file pone.0154410.s001.docx]

**Supplementary File (S1 File)**

**Table A. Ingredients and nutrient composition of broiler diets, as-fed basis.**

| **Items** | **1 to 21 d** | **22 to 42 d** |
| --- | --- | --- |
| **Ingredient (g/kg)** |  |  |
| Maize | 570.0 | 621.7 |
| Soybean meal | 310.0 | 231.0 |
| Corn gluten meal | 40.0 | 60.0 |
| Soybean oil | 30.0 | 40.0 |
| Dicalcium phosphate | 20.0 | 16.0 |
| Limestone | 12.0 | 14.0 |
| L-Lysine | 3.5 | 3.5 |
| DL-Methionine | 1.5 | 0.8 |
| Salt | 3.0 | 3.0 |
| Premix^1^ | 10.0 | 10.0 |
| Total | 1000 | 1000 |
| **Calculated Nutrition levels^2^** |  |  |
| AME (MJ/kg ) | 12.57 | 12.97 |
| Crude protein | 214.9 | 196.3 |
| Calcium | 9.9 | 9.5 |
| Available Phosphorus | 4.6 | 3.9 |
| Lysine | 12.1 | 10.45 |
| Methionine | 5.1 | 4.2 |
| Methionine + cysteine | 8.6 | 7.6 |
| **Analysed composition^3^** |  |  |
| Crude protein | 210.4 | 198.7 |
| Ash | 54.9 | 51.5 |

*Note.* ^1^Premix provided per kg of diet: limestone, 3.3 g; l-lysine HCl, 1.5 g; dl-methionine, 1.3 g; VA 10,000 IU, VD3 3000 IU, VE 30 IU, menadione, 1.3 mg, thiamine 2.2 mg, riboflavin, 8 mg, nicotinamide 40 mg, choline chloride 600 mg, calcium pantothenate 10 mg, pyridoxine HCl, 4 mg, biotin 0.04 mg, folic acid 1 mg; vitamin B12 (cobalamine) 0.013 mg, Fe (from ferrous sulphate) 80 mg, Cu (from copper sulphate) 8 mg, Mn (from manganese sulphate) 110 mg, Zn (Bacitracin Zn), 65 mg, iodine (from calcium iodate) 1.1 mg, Se (from sodium selenite), 0.3 mg. ^2^The nutrient levels were on an as-fed basis. ^3^Values based on analysis of triplicate samples of diets.

**Table B. Effects of different treatments on development of jejunum of broilers chickens.**

|  | **Item^1^** | **CON ^2^** | **SB ^3^** | **CLI ^4^** | **CLI-B ^5^** |
| --- | --- | --- | --- | --- | --- |
| **21 d** | Relative weight (g/kg BW) | 17.31 ± 0.48 | 17.67 ± 0.79 | 18.09 ± 0.68 | 18.60 ± 0.74 |
|  | Relative length (cm/kg BW) | 88.56 ± 1.91 | 91.09± 5.75 | 88.39± 3.20 | 91.13± 3.26 |
| **42 d** | Relative weight (g/kg BW) | 9.47 ± 0.20 | 9.69 ± 0.51 | 9.27 ± 0.19 | 9.90 ± 0.31 |
|  | Relative length (cm/kg BW) | 26.07 ± 1.06 | 25.10 ± 0.58 | 27.13 ± 0.57 | 27.48 ± 0.98 |

*Note.* ^1^Abbreviations: BW, body weight. ^2^ Broilers fed a basal diet. ^3^ Broilers fed a basal diet supplemented with 0.05% sodium butyrate. ^4^ Broilers fed a basal diet supplemented with 1% clinoptilolite. ^5^ Broilers fed a basal diet supplemented 1% CLI-B. ^ab^ Means within a row with different letters (a, b) differ significantly (P < 0.05).
